# Survey of general practitioners’ awareness, practice and perception of social prescribing across Europe

**DOI:** 10.1080/13814788.2024.2351806

**Published:** 2024-05-17

**Authors:** Sinah Evers, Joyce Kenkre, Thomas Kloppe, Donata Kurpas, Juan M. Mendive, Ferdinando Petrazzuoli, Josep Vidal-Alaball

**Affiliations:** a Department of Health Services Research, Institute of Public Health and Nursing Research, University of Bremen, Bremen, Germany; b Health Sciences Bremen, University of Bremen, Bremen, Germany; c WONCA Europe Social Prescribing and Community Orientation Special Interest Group, Brussels, Belgium; d Life Sciences and Education, University of South Wales, Pontypridd, UK; e Department of General Practice and Primary Care, University Medical Centre Hamburg-Eppendorf, Hamburg, Germany; f Health Sciences Faculty, Wroclaw Medical University, Wroclaw, Poland; g European Rural and Isolated Practitioner Association (EURIPA), Paris, France; h La Mina Primary Health Care Academic Centre, Catalan Health Institute (ICS), University of Barcelona, Barcelona, Spain; i Institute for Primary Health Care Research IDIAP Jordi Gol, Barcelona, Spain; j Department of Clinical Sciences in Malmö, Centre for Primary Health Care Research, Lund University, Malmö, Sweden; k Health Promotion in Rural Areas Research Group, Institut Català de la Salut, Barcelona, Spain; l Central Catalonia Research Support Unit, Jordi Gol i Gurina University Institute for Research in Primary Health Care Foundation, Sant Fruitós de Bages, Barcelona, Spain; m Faculty of Medicine, Vic-Central University of Catalonia, Vic, Spain

**Keywords:** Social prescribing, general practice, Europe, complex intervention, healthcare system

## Abstract

**Background:**

Social prescribing (SP) is a patient pathway by which healthcare professionals connect patients with other sources of support, groups, or activities within their community. The awareness, practice, and perception of SP among GPs across Europe remains unclear.

**Objectives:**

To explore the awareness, practice, and perception of GPs on SP in the WONCA Europe region.

**Methods:**

An anonymous, cross-sectional online survey was distributed through a snowballing system, mailing lists, and at three international conferences in 2022/2023 to explore GPs’ awareness, practice, and perception of SP. The questionnaire in English contained 21 open and closed questions.

**Results:**

Of the 208 participating GPs from 33 countries, 116 (56%) previously heard of ‘social prescribing’ and 66 (32%) regularly referred patients to community activities through a formal system. These 66 GPs reported different funding sources and varied activities, with an average of four activities and physical exercise being the most prevalent. Among them, 25 (38%) knew about national or local SP awareness campaigns. Of these 25, 17 (68%) agreed that SP increases their job satisfaction and 21 (84%) agreed that it has a positive impact on their patients. Variations in SP awareness and referral practice were evident across and within countries.

**Conclusion:**

Despite disparities in awareness and referral practice as well as a diversity of activities and funding sources, most GPs who actively referred patients and were informed about SP campaigns agreed that SP positively impacts them and their patients.

## KEY MESSAGES

Knowledge of social prescribing differs among and within countries.

A third of general practitioners reported they regularly refer their patients through a formal system to access activities and groups in the community.

General practitioners agree that social prescribing increases job satisfaction and positively impacts patients’ health and well-being.

## Introduction

Social prescribing (SP) is a patient pathway by which healthcare professionals connect patients with non-medical sources of support, social groups, or activities to improve their health and well-being [1,2]. The design of SP programmes varies significantly [3]. Still, they typically involve a link worker who supports patients in identifying their needs and connects them with appropriate community-based support, activities, or groups [1]. The approach recognises that health and well-being are influenced by various social, economic and environmental factors [4–6].

Due to the variable implementation of SP, targeted outcomes and their evaluation are diverse. On an individual level, the focus often lies on improved well-being and reduced loneliness, aiming to enhance the person’s health in the long term [2]. At the community level, targets can be to strengthen the sense of belonging or to identify and utilise resources [2]. On a systemic level, the utilisation of healthcare services, such as primary care consultations or hospital admissions, is intended to be reduced [2]. Nevertheless, the evidence base is still developing [7–9].

The United Kingdom (UK) was the first country to implement SP on a national scale as part of its national health strategy. The aim was to provide general practitioners (GPs) and other medical staff with expanded options, enabling them to refer patients to existing sources of support, groups, or activities [10].

While some GPs already refer some patients to external support for social problems [11], the recent shift towards a more structured integration of health care and social support systems is a notable development. Additionally, introducing specialised roles such as link workers, dedicated to streamlining these connections, represents an innovative change in this field. The holistic concept of SP has gained increasing interest across Europe, with different models and approaches being implemented in various countries [12–15].

By recognising and incorporating social and environmental factors, SP creates more holistic healthcare systems [16]. Given that GP engagement is crucial for SP implementation, understanding their current practice and perception of it is vital. This study aims to capture GPs’ awareness, practice, and perception of SP across Europe by exploring their perspective on their own understanding, activities, funding, and the impact of SP.

## Methods

### Study design

This anonymous, cross-sectional online survey was designed to capture the awareness, practice, and perception of GPs regarding SP in the WONCA Europe region.

### Questionnaire development

The initial questionnaire draft was developed based on research objectives and an extensive literature review. The authors then assessed the face validity and length to ensure relevance and accuracy. It contained 21 open and closed questions in English and was tailored to be responded to by GPs.

In the questionnaire, the participants were initially presented with an explanation of what SP is (see Supplementary File 1: questionnaire). The question format included five yes/no questions, such as whether participants had previously heard of the term SP. Four questions used a Likert-type scale, allowing participants to rate their responses on a scale of 1–5. There were two open-ended questions, one inquiring about specific terms used for SP in the participant’s country. Three questions permitted multiple responses, covering topics like associated SP activities or funding sources. Additionally, there was one multiple-choice question for participants to select the most applicable option. The demographic section consisted of six questions: four multiple-choice questions regarding gender, country, practice location, and job title, and two numerical entry questions for age and years in practice. Nine questions were mandatory for respondents to answer.

Our survey enquired whether GPs routinely used a formal pathway to refer patients to community activities and groups, with the options ‘yes’ and ‘no’. Only those answering ‘yes’ were included in the latter part of the survey, as this ‘formal pathway’ was categorised as an engagement similar to SP.

### Data collection

The questionnaire was shared *via* Google’s online survey tool. A snowballing method was used to disseminate the survey across the authors’ networks from May 2022 to February 2023. Additionally, it was shared in 2022 at the EURIPA Forum in Catania, Italy, at the WONCA World Rural Health Conference in Limerick, Ireland and the WONCA Europe Conference in London, UK. Further, it was sent to the 46 national member organisations of the WONCA Europe and they were asked to send it to their member organisations or individual members *via* e-mail in 2022. Data was collected between 23 May 2022 and 19 February 2023. Due to the different recruitment strategies, the response rate is unknown.

### Data analysis

Following data collection and cleaning, numerical responses underwent descriptive analysis, while free-text responses were examined and categorised into themes. Owing to the limited sample size, statistical significance was not assessed. The results section presents a narrative summary of the data, supplemented by selected Tables and Figures.

## Results

### Participant characteristics

Of the 240 participants, 32 were excluded (non-GPs or outside of the WONCA Europe region), leaving participants from 33 countries (Table 1).

**Table 1.** Participants’ countries of practice.

Country	Participants		
Austria	2	Malta	1
Belgium	5	Montenegro	1
Bulgaria	2	Netherlands	2
Croatia	13	Norway	1
Cyprus	2	Poland	2
Czech Republic	4	Portugal	12
Denmark	2	Romania	5
France	14	Serbia	1
Georgia	3	Slovakia	2
Germany	10	Slovenia	2
Greece	2	Spain	17
Hungary	3	Sweden	1
Ireland	7	Switzerland	17
Israel	14	Turkey	20
Italy	27	Ukraine	1
Latvia	3	United Kingdom	8
Macedonia	2	*n*	208

Table 2 shows the characteristics of all 208 participants, including age, gender, years of practice, and their practice location.

**Table 2.** Participant characteristics.

Participant characteristics	*N*	%
*n*	208	
Age		
25–34	39	18.8
35–44	53	25.5
45–54	48	23.1
55–64	42	20.2
≥65	26	12.5
Gender		
Female	122	58.7
Male	85	40.9
Prefer not to say	1	0.5
Years of Practice		
0–9	53	25.5
10–19	55	26.4
20–29	42	20.2
≥30	58	27.9
Practice location		
Rural	36	17.3
Semi-rural	54	26
Urban	118	56.7

One value is missing due to one participant not answering one question. Participant numbers fell from 208 to 66 after the first filter question. A second filter, on awareness of SP campaigns, further reduced the count to 25.

### Awareness of social prescribing

**Figure 1.**
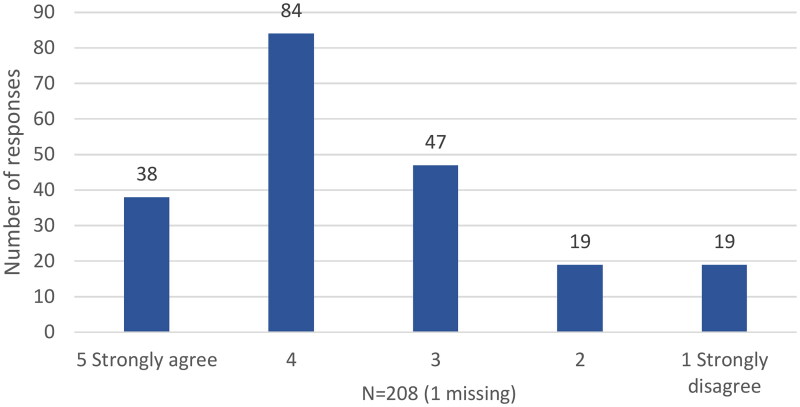
GPs agreement with the statement ‘I understand what social prescribing is’.

Of the 208 GPs, 116 (56%) had previously heard the term ‘social prescribing’. Additionally, 122 (59%) agreed they understood what SP is, with one missing response (Figure 1).

When asked if alternative terms were used in their own countries to refer to a similar concept to SP, most respondents indicated that there was no specific term or that they used the term ‘social prescribing’. However, some mentioned different terms for SP that focus on particular aspects, such as exercise or nature on prescription. Most other terms were similar to the English expression, for example, ‘Prescrição Social’ (Portugal), ‘Prescrizione Sociale’ (Italy), ‘Prescripción social’ (Spain), ‘Welzijn op Recept’ (Netherlands), ‘welzijn op voorschrift’ (Belgium), or ‘soziales Rezept’ (Germany).

**Figure 2.**
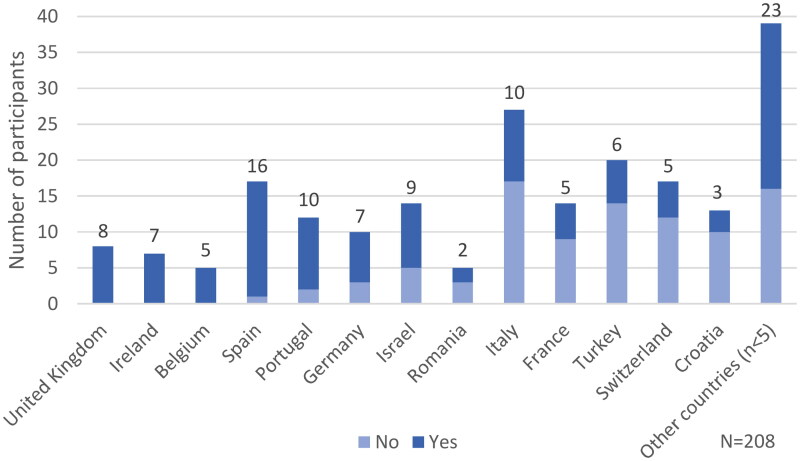
Distribution of GPs across European countries who heard the term social prescribing before.

In the countries with at least five participants, between 23% and 100% of the GPs stated they had heard of SP previously, with the term being known by all in the UK, Ireland and Belgium (Figure 2).

Among rural GPs, 27 (75%) responded affirmatively to having heard of SP, compared to 23 (43%) of the GPs from semi-rural areas and 66 (56%) in urban areas.

### Practice of social prescribing

Among the participants, 66 of 208 (32%) reported that they regularly referred their patients through a formal system to access activities and groups in the community. In comparison, 142 (68%) reported that they did not.

**Figure 3.**
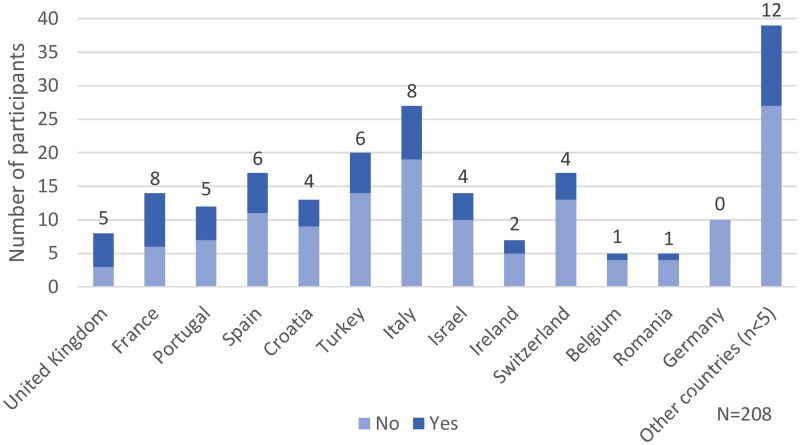
Distribution of GPs across European countries who routinely refer their patients through a formal system to access activities and groups in the community.

The variation among countries extends to whether GPs routinely refer their patients to community activities and groups through a formal system (Figure 3). In all countries except Germany, with more than five participants, the proportion of GPs answering ‘yes’ ranged from 20% to 63%, with an average of 31%.

A wide range of services and activities were included in the various SP pathways. The activity cited most often was physical exercise by 58 of the 66 participants, followed by social counselling (40), arts and crafts (32), leisure groups (30), green gyms (29), welfare advice (28), other cultural activities (24), education on prescription (19), books on prescription (14), and other (3). On average, each participant named four different activities.

**Figure 4.**
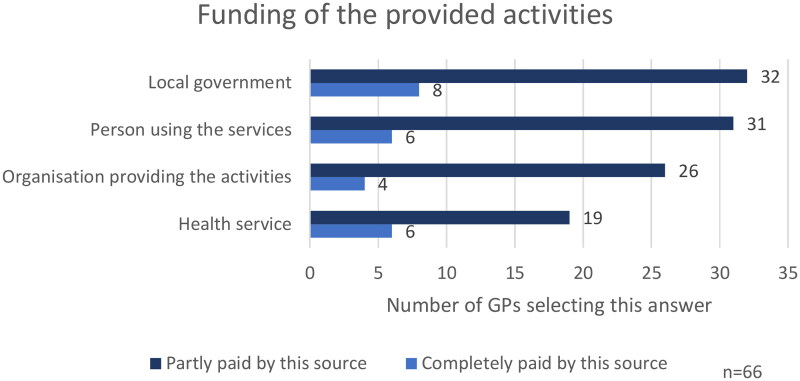
GPs perception of funding sources of social prescribing activities.

SP was often funded through multiple sources. The study found that 24 (36%) of 66 participants named a single funding source, while most named numerous funding sources. The distribution of funding sources was evenly spread, which included the person using the service, the organisation providing the activity, the local government, and the health service, as depicted in Figure 4. Participants could choose one or multiple options: the darker bar shows those selecting an option among others, while the lighter bar shows those choosing it exclusively. For example, six participants (9%) said the health service fully funds SP, whereas 23 also chose additional funding sources.

On enquiry about the financial side of SP from the patient’s perspective, of the 66 GPs, 31 (47%) stated that activities were ‘free of charge’, followed by 15 (23%) stating ‘paid partially by the patient’, 8 (12%) ‘paid fully by the patient’, and 12 (18%) stated it ‘depends’.

In terms of healthcare professionals receiving payment for their SP referrals, 6 (9%) of 66 respondents answered yes, while 58 (88%) said no and 2 (3%) were unsure. In contrast, 21 (32%) of the GPs reported link workers received payment for their referrals.

### Perceived impact of social prescribing

**Figure 5.**
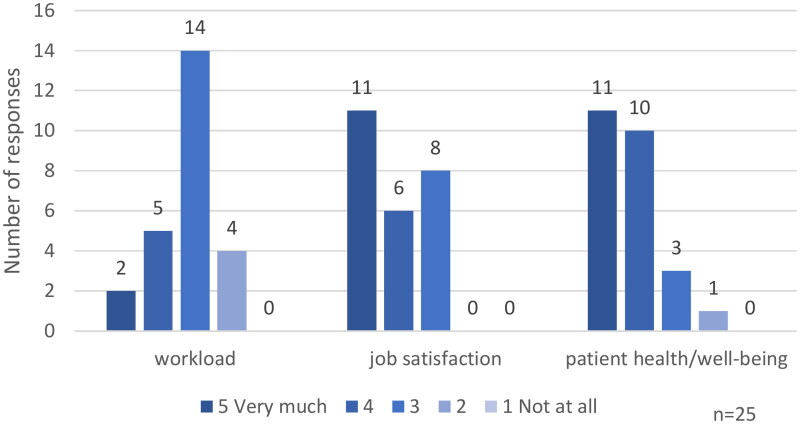
Perceived impact of SP on workload, job satisfaction and patient health/well-being by GPs.

Twenty-five (38%) of 66 GPs stated they had local or national projects to improve awareness of SP. They were asked for their perception of the impact of SP on their workload, job satisfaction and patient well-being. Out of these 25 GPs, 17 (68%) agreed that SP increases their job satisfaction, while 21 (84%) agreed that SP has a positive impact on their patient’s health and well-being (Figure 5).

## Discussion

### Main findings

About 56% of GPs had heard of ‘social prescribing’ and 59% said they understood it. A third of GPs regularly referred patients to community activities through a formal system. Awareness and practice varied by region. Most of the asked GPs reported increased job satisfaction and a positive impact on patient health and well-being by SP.

### Awareness of social prescribing

This study provides insight into the awareness, practice, and perception of GPs regarding SP across Europe. It was surprising that more than half of GPs had previously heard of the term ‘social prescribing’, considering it is an English language expression and a concept that does exist to a very different degree in current healthcare systems. The proportion of GPs that rate their understanding of the SP concept highly was notable, with 122 (59%) expressing confidence in their knowledge. This is unexpected, given how different SP concepts include target groups and outcome parameters [2]. Even within the UK, where there is a national objective to implement SP, the knowledge about and understanding of SP varies and many GPs still have misconceptions [17].

When asked about their familiarity with the term SP, the responses from GPs within different countries revealed variations. As expected, participants from the UK and Ireland all had heard the term before.

In conclusion, over half were already familiar with the term and, after an explanation, about half claimed to understand SP well. Along with the varying familiarity with the term across countries, this suggests varying awareness within and among countries.

### Practice of social prescribing

A diversity of responses on referring to community activities and groups across different countries was notable, indicating no clear pattern in referral practices to support services, social groups, or activities. Instead, the decision to refer a patient often depends on the individual GP. These findings may suggest that personal motivation and local structures are crucial in whether patients are referred. This underscores the variability not only among but also within countries.

The range of services and activities GPs associated with SP varied, with an average of four options cited, demonstrating an understanding that SP encompasses more than one service. This reflects an awareness among GPs of the diverse support SP can offer and highlights the critical role of flexibility in SP programmes. Revealing the GP’s recognition of the comprehensive nature of SP and the wide array of options to address varied needs within communities.

The answers given regarding the funding of SP indicate that there are no fixed responsibilities in place or that GPs do not know of them. GPs usually did not receive financial reimbursement for connecting patients to other sources of social support, even though doing so usually requires their time to gain knowledge about available resources as well as their time in referring the patient. Further, two-thirds of the link workers were said not to be paid for their services. This is likely due to the integrations of volunteers in some instances. This fits with SP commonly encountering funding difficulties [18]. One contributing factor to this challenge is that SP operates across sector boundaries, complicating the allocation of funding responsibilities. In the UK, however, where SP funding has been politically designated as a National Health Service responsibility, the financial obligations for link workers are covered [18]. Nevertheless, even there, the costs for third-sector services are rarely included [18].

Based on the variations in activities and funding, indicating different local structures, it will be crucial to develop a shared language (across languages) and a well-defined framework for SP while still allowing sufficient flexibility for local and individual adaptations.

### Perceived impact of social prescribing

The results indicate that GPs who frequently refer their patients *via* formal pathways and are aware of SP campaigns in their country expected positive effects from SP, especially by enhancing job satisfaction and benefiting their patients.

However, practical experiences indicate that engaging GPs effectively is challenging [17]. Therefore, including them in the planning and implemention of SP programmes is crucial, along with patients and other professionals. A collaborative approach that engages all stakeholders is essential for the successful integration and optimisation of SP services.

### Strength and limitations

A strength of our study is the wide-ranging participation of actively practising GPs from across Europe, offering insights that closely align with the realities of professionals engaged in frontline healthcare. Additionally, the diverse backgrounds of our authors constitute a strength, covering a variety of professions and countries.

Limitations include the low number of responses within each country with considerable differences among countries. Therefore, this study is not representative. Because a participant’s choice for participation was based on motivation, a selection bias is probable, leading to a sample of GPs who is more motivated or more interested in the topic.

Our questionnaire was refined in several group discussions and developed by consensus. Nonetheless, it was not validated against other measures besides a face validation procedure.

### Implications

While the nuanced, small-scale context plays a crucial role in the implementation of SP, it is noteworthy that similar challenges are encountered across European countries. This shared experience highlights the critical need for an active exchange of insights and the facilitation of knowledge transfer among countries.

## Conclusion

The present study provides valuable insights into the perception of GPs in Europe regarding SP. It highlights the diversity across and within countries in terms of awareness of the SP concept and the practice of regularly referring patients to community activities and groups. Despite these variations, as well as the perception of a wide range of activities and differences in funding, GPs who frequently made referrals and knew of local or national awareness campaigns tended to perceive SP to have a positive impact on them and their patients.

## Supplementary Material

Supplemental Material
