# Supplementary material for: Survey of general practitioners’ awareness, practice and perception of social prescribing across Europe
Source: Eur J Gen Pract. 2024 May 17;30(1):2351806. doi: 10.1080/13814788.2024.2351806 (PMC11104702; doi:10.1080/13814788.2024.2351806)
Supplement: Supplemental Material [file IGEN_A_2351806_SM8264.pdf]

1. General information and knowledge about social prescribing

Social prescribing (SP) is a way of linking patients in primary care with sources of support to improve their health and wellbeing within their community. For example, if a health care worker identifies loneliness, stress or weight gain in a patient, they can connect him/her to other sources of support. They may make the referral directly to groups/activities in their community (e. g. counselling, outdoor activities, creative groups, physical activities or support groups) or they may refer to a 'coordinator' / link worker to make the referral.

Social prescribing programmes are being widely promoted in the UK and are now being increasingly adopted all over Europe but, nevertheless, formats differ, since social prescribing is highly dependent on the context, for example the health care system and facilities in the country/region.

Consequently, there is confusion about what constitutes social prescribing and, moreover, it is sometimes not immediately understood by health care professionals themselves. Therefore, this proposed study aims to map across Europe established social prescribing programmes and how they are organised. This will assist in establishing a clear definition of "social prescribing" for Europe and greater understanding within communities. The questionnaire will take you less than 10 minutes to complete. Thank you very much in advance for your cooperation.

The Social Prescribing and Community Orientation Working Group

1. Have you ever heard of the term social prescribing? *

☐ Yes
☐ No

2. How would you agree with the statement: "I understand what Social prescribing is."

☐ 1 Strongly disagree
☐ 2
☐ 3
☐ 4
☐ 5 Strongly agree

3. Taking into account the explanation above, can you tell us what term is used to define Social prescribing in your country? (In your own language with the translation in English)*

[ ]

4. I routinely refer my patients, through a formal system, to access activities and groups in the community*

☐ Yes
☐ No

2. Organisation and activities related to social prescribing

5. How does social prescribing work in your country? Please tick all that apply.

☐ Nationally
☐ Regionally
☐ Locally
☐ Individual Practice
☐ Individual Family Doctor/GP
☐ Other: ________________

* required

6. If social prescribing exists in your country, what social services, activities, and other support are associated with it?
☐ Arts on Prescription
☐ Books on Prescription
☐ Other Cultural Activities
☐ Education on Prescription
☐ Physical Exercise
☐ Green Gyms (becoming physically and mentally healthier through contact with nature gardening, walking in parks, etc.)
☐ Welfare Advice
☐ Social Counselling
☐ Leisure groups
☐ Other: ________________

7. Do you have any local or national projects to improve awareness on social prescribing?
☐ Yes
☐ No
☐ I don't know

8. If yes, please describe

2. Financial aspects

9. Who is funding the activities provided? Please tick all that apply.
☐ Health Service
☐ Local government
☐ Paid for by the organisation providing the activities
☐ Paid for by the person using the services

10. How does it work for the patient?
☐ Paid fully by the patient
☐ Partially paid for by the patient
☐ Free of charge
☐ Other: ________________

11. Are the health care professionals (eg family doctors, nurses) paid for their social prescribing referrals?
☐ yes
☐ no
☐ Other: ________________

12. Is the 'Link Worker' paid for his/her social prescribing activity?
☐ yes
☐ no
☐ Other: ________________

3. Impact of social prescribing

	1 Not at all	2	3	4	5 Very much
13. How much positive impact do you think social prescribing has on your patients' health and wellbeing?	☐	☐	☐	☐	☐
14. How does social prescribing affect your workload?	☐	☐	☐	☐	☐
15. What impact do you think social prescribing has on your job satisfaction?	☐	☐	☐	☐	☐

4. Demographic information

16. Your gender *

☐ Male
☐ Female
☐ Other
☐ I prefer not to say

17. Your age *

[ ]

18. Country you practise in: *

Choose from the List: ☐

19. Where do you practice? (You can choose only one answer) *

☐ Urban
☐ Semirural
☐ Rural

20. What's your job? *

☐ doctor
☐ nurse
☐ social worker
☐ Other: _________________

21. How many years have you been practicing? *

[ ]

* required
